# Intelligent Generation Method of Innovative Structures Based on Topology Optimization and Deep Learning

**DOI:** 10.3390/ma14247680

**Published:** 2021-12-13

**Authors:** Yingqi Wang, Wenfeng Du, Hui Wang, Yannan Zhao

**Affiliations:** 1 Institute of Steel and Spatial Structures, College of Civil Engineering and Architecture, Henan University, Kaifeng 475004, China; WangYingqi666666@outlook.com (Y.W.); pkujx06885098@outlook.com (H.W.); ZhaoYannan666@outlook.com (Y.Z.); 2 Henan Provincial Research Center of Engineering Technology on Assembly Buildings, Kaifeng 475004, China

**Keywords:** innovative structures, topology optimization, boundary equilibrium generative adversarial networks, additive manufacturing, material consumption

## Abstract

Computer-aided design has been widely used in structural calculation and analysis, but there are still challenges in generating innovative structures intelligently. Aiming at this issue, a new method was proposed to realize the intelligent generation of innovative structures based on topology optimization and deep learning. Firstly, a large number of structural models obtained from topology optimization under different optimization parameters were extracted to produce the training set images, and the training set labels were defined as the corresponding load cases. Then, the boundary equilibrium generative adversarial networks (BEGAN) deep learning algorithm was applied to generate numerous innovative structures. Finally, the generated structures were evaluated by a series of evaluation indexes, including innovation, aesthetics, machinability, and mechanical performance. Combined with two engineering cases, the application process of the above method is described here in detail. Furthermore, the 3D reconstruction and additive manufacturing techniques were applied to manufacture the structural models. The research results showed that the proposed approach of structural generation based on topology optimization and deep learning is feasible, and can not only generate innovative structures but also optimize the material consumption and mechanical performance further.

## 1. Introduction

As one of the most remarkable achievements in the field of structural engineering, computer-aided design has been widely used in the calculation and analysis of various engineering structures, which has not only significantly improved the accuracy and efficiency of structural calculations but also solved the analysis and design problems of complex structures that have been difficult to complete manually in the past [1]. However, until now, the design of practical engineering structures has still mainly depended on the designers’ experiences and intuition in the design process. Designers carry out conceptual design and propose a preliminary model at first. Then, they apply a computer to aid in calculation and analysis. Finally, model adjustment is carried out before determining the final design scheme. The above process is usually cyclic and repeated several times, and model tests must sometimes be conducted. Therefore, artificial design usually has a long cycle time and high energy consumption, especially since the designers’ level of experience can greatly influence the design quality. In the stage of conceptual design, designers can rely only on experience and imagination to build the initial model. However, with the development of modern engineering technology, the structural system is getting larger and more complex, and it can be difficult for designers to finish the optimal design depending on their experience [2]. Using a computer to automatically generate innovative and optimal structures to enhance the intelligence of structural design, further shorten the design cycle, and improve the design quality has become one of the development trends of structural design [3].

In response to this challenging problem, scholars have conducted a large number of research explorations, which can be divided into two schools of thought. One is to explore the problem from the perspective of derivative design. Balachandran [4] proposed a knowledge-based computer-aided design (CAD) integrated system. The system was applied in architectural design, and it could automatically verify whether a design met specific requirements through the description in the CAD database. Zhao et al. [5] programmed the performance-based structure generation algorithm structural topology and shape annealing (STSA), which applies computational synthesis theory to numerically realize the intelligent generation and conceptual design of spatial trusses. Engineers from General Motors Company and Autodesk collaborated to generate more than 150 feasible design alternatives for seat brackets utilizing derivative design, and eight different components were assembled into one integrated part that achieved a weight reduction of 40% and a strength increase of 20% [6]. Lightning Motorcycles and European Aerospace also explored the derivative design for motorcycle swing arms and aircraft cabin bulkheads, respectively, to achieve weight reduction while maintaining strength [7].

The other school of thought is to explore the problem from the perspective of topology optimization. Topology optimization integrates structural design with mathematical optimization theory. It converts the problem of finding the optimal topology of a structure into the problem of finding the optimal material distribution in a given designable area. Representative topology optimization algorithms, such as bidirectional evolutionary structural optimization (BESO), smooth-edged material distribution for optimizing topology (SEMDOT), and solid isotropic material with penalization model (SIMP) are widely used in engineering fields, including machinery, aerospace, auto, and construction [8,9,10]. However, the current topology optimization algorithms require pre-specification of the designable area, setting of the optimization objectives, and determination of the loads and constraints, so the human workload is still large [11]. Moreover, the existing topology optimization design produces only the optimal design within the framework of the established designable area. Only a single optimal solution is generated, regardless of whether the solution is practical and elegant or not. It provides designers with limited control to modify the optimization model output [12]. Therefore, it is still worth exploring better structural solutions from a broader perspective, including design diversity and aesthetics [13].

Judging from the progress of the above two schools of thought, it is still a considerable challenge to achieve the goal of intelligent generation of optimal structure. In recent years, artificial intelligence (AI) has developed very rapidly and presents great potential value in structural intelligence generation [14,15,16]. Sosnovik et al. [17] first applied deep learning in the field of topology optimization by transforming the topology problem into an image segmentation problem, producing pixel-level structurally-labeled images and generating topology results using convolutional neural networks with good generalization capabilities and significant acceleration performance. Lee et al. [18] proposed the use of convolutional neural networks instead of the finite element method to calculate compliance. Taking one messerschmitt-bölkow-blohm (MBB) beam and two cantilever beam problems as examples, the approach used convolutional neural networks to capture and train images of topological structures and accelerate the batch processing of data by graphics processing unit (GPU), which proved the applicability and robustness of the method. Lin et al. [19] proposed combining the traditional SIMP method with deep learning to accelerate the topology optimization of thermally conductive materials. The optimization results were fed into an encoding–decoding fully convolutional network (FCN) algorithm to obtain material layouts with high thermal conductivity, which significantly reduced the time consumption of the optimization process. Li et al. [20] built up a deep learning method of non-iterative topology optimization based on generative adversarial network (GAN) and super-resolution generative adversarial network (SRGAN). This method attempted to directly predict the approximate optimal structure under different boundary conditions and generate accurate topological structures of the conductive heat transfer by training the artificial neural network, which significantly reduced the computational power of topology optimization. Yu et al. [21] used conditional generative adversarial network (CGAN) to improve the resolution of topological results under a given set of boundary conditions and optimization parameters. A near-optimal structure was determined by supervised learning of a dataset of optimized structures, and there was no iterative computation in the generation process. Sim et al. [22] proposed a new topology optimization method based on GANs and clustering analysis. A topology optimization validation curve (TOVC) was applied to obtain the optimized valid data by clustering analysis. It was able to solve the time-consuming problems of topology optimization and generate better solutions. Abueidda et al. [23] developed three convolutional neural network (CNN) models to predict optimal design. The topology optimization framework was utilized for training and verifying the CNN model. The CNN models could accurately predict the optimal configuration with little calculation time. Ates et al. [24] proposed a two-stage convolutional encoder–decoder network model that incorporated a new loss function approach to reduce the number of structural disconnection cases and pixel errors. It promoted the topology-optimized prediction performance of deep neural networks (DNNs) without any iterations, while improving volume fraction errors and compliance. Behzadi et al. [25] proposed a CGAN with the knowledge capability to transfer learning methods for generative design. The algorithm significantly reduced the scale of the training set and improved the topological connectivity of the predicted structures. Chi et al. [26] presented a topology optimization architecture based on machine learning. The framework was highly scalable and it rapidly managed topology optimization difficulties with different design considerations. Moreover, it efficiently solved large-scale topology optimization problems while maintaining accuracy. Halle et al. [27] introduced an artificial intelligence-assisted topology optimization design method that did not require pre-optimized data. The technique significantly reduced the computational effort and provided a new method to solve problems that required difficult or unavailable data. Nie et al. [28] put forward a data-driven TopologyGAN model. The model was able to reduce the mean square and absolute errors for test problems involving previously undiscovered boundary conditions. A hybrid network was also introduced for the generator to improve the overall accuracy. Qian et al. [29] proposed double-model artificial neural networks for replacing the topology optimization model. An efficient data generation method applicable to topology optimization design was also developed, which saved training time and sped up the design process of topology optimization. Zhang et al. [30] developed a deep CNN model with powerful production capability for topology optimization. This method significantly reduced the computational cost without sacrificing the optimality of the design solution. In addition, it could deal with the problem of boundary conditions not included in the training dataset. The analysis of scholars’ current research status and development trends shows that applying AI deep learning technology to achieve the intelligent generation of innovative structures is quite promising. However, existing structural intelligent generation methods do not comprehensively consider topology optimization parameters, resulting in fewer varieties and poor processability of developed models. In particular, the discussion of the mechanical properties and evaluation methods of the generated solutions is very lacking. Therefore, the current research methods are not universally applicable in practical engineering applications.

The method proposed in this paper integrates the parameters of manufacturing process constraints and evaluates the generated solutions to help in solution decision-making. It can develop many innovative, aesthetically pleasing, and machinable schemes with good mechanical properties. The basic idea and correlative theories of this method are firstly introduced in the second section. Then, the specific implementation process of this method is explicitly discussed in the third section. Sections four and five complete two examples to demonstrate the feasibility of the structural intelligent generation method. Moreover, the integrated manufacturing of the two examples is realized with the help of 3D reconstruction and additive manufacturing technology.

## 2. Basic Idea and Correlative Theories

### 2.1. Basic Idea

**Figure 1:**
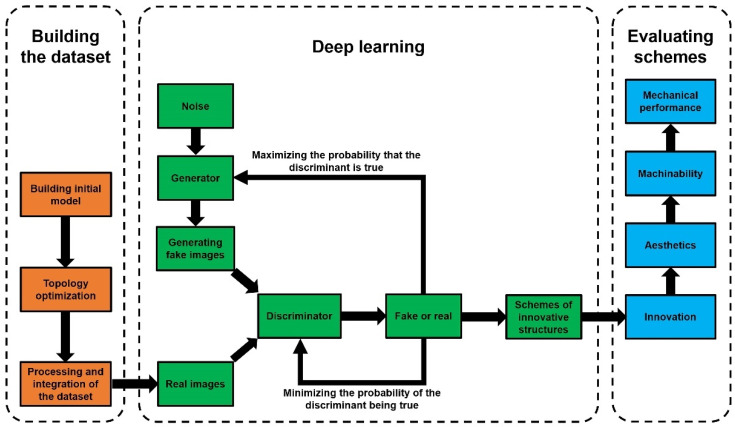
The technical route for the intelligent generation method of innovative structures.

The basic idea of the proposed method is to apply the deep learning techniques of AI to achieve the intelligent generation of innovative structures. In essence, it is the computer, instead of designers, that learns from the experience accumulated from the past data at first. Then, the deep learning algorithm is used to generate innovative structures. There are two critical scientific issues. One is the issue of how to build a learning library with a large number of samples as the basis of deep learning. In this paper, we applied the topology optimization method to obtain abundant optimized structural models under different element density values, different constraints, and different load cases to build a dataset. This method not only provides numerous learning samples to solve the problem of making a model library with a large volume of data but can also inherit the advantage of high material utilization from topology optimization models to improve the quality and efficiency of deep learning. The other critical scientific issue is the evaluation of generated innovative structures. Since the number of generated structural models is numerous, an evaluation system is necessary to evaluate the generated results and select the optimal scheme. The technical route for the intelligent generation method of innovative structures is shown in Figure 1.

(1) During the dataset building stage, an initial model is first built and divided into designable and non-designable areas. Then constraint parameters, such as checkerboard control, penalty factor, and minimum member size, are considered in the topology optimization calculation. The topology optimization results are enriched by adjusting the element density thresholds and load case types. Eventually, all images of topology optimization models with different optimization parameters are collected as training set images, and training set labels are defined as the corresponding load case types.
(2) During the deep learning stage, the BEGAN algorithm is programmed based on the open-source deep learning framework TensorFlow. Two sub-models of the BEGAN, named the generator model and the discriminator model, are set up. The generator is responsible for learning the data distribution and generating examples similar to the input samples. In contrast, the discriminator is responsible for distinguishing the actual samples from the generated fake samples. The generator tries to cheat the discriminator, while the discriminator tries not to be tricked by the generator. Both the generator and the discriminator are alternately optimized and trained to improve. Eventually, they reach Nash equilibrium and generate optimal design schemes of innovative structures.
(3) During the evaluating schemes stage, an innovative representative structure from the results is selected according to the evaluation indexes for comparative argumentation and analysis. The 3D model of the chosen example is built up via 3D reconstruction technology for images, and the structural model is 3D printed by applying additive manufacturing technology.

### 2.2. Topology Optimization Theory

The SIMP density-stiffness interpolation model was used to establish the topology optimization mathematical model of the initial model. The design variable is defined as the element density of each element in the designable area of the finite element model, which is related to the material elastic modulus and takes a value ranging from 0 to 1; thus, the topology optimization problem of the structure is transformed into the issue of solving for the optimal material distribution [31]. If the material is set to be isotropic, Poisson’s ratio is associated with the elastic modulus of the structure and does not change with the relative density of elements [32]. The formula of the elastic modulus and element density can be established as:

(1) $$E\left(x_{i}\right)=E_{min}+x_{i}^{p}\left(E_{0}-E_{min}\right)$$

where $x_{i}$ is the relative element density; p is the penalty factor, which penalizes the intermediate density values and reduces intermediate density elements; and $E_{min}$ and $E_{0}$ are the elastic moduli of the material in the regions of element relative density of 0 and 1. When $E_{min}=E_{0}/1000$, stiffness matrix singularities are avoided.

An approximate model was established using the optimization criterion method based on the SIMP method, with the volume fraction of the designable area as the constraint and the maximized stiffness of the optimized region as the optimization objective [33], which can be expressed in mathematical language as:

(2) $$\left\{ \begin{array}{c} \mathrm{Find}:\;\;\;\;\;\;\;\;\;\;\;\;x={\left(x_{1},x_{2},\cdots ,x_{n}\right)}^{T}\\ \mathrm{Min}:\;\;\;\;\;\;\;\;\;\;\;\;\;C\left(x\right)=\frac{1}{2}U^{T}KU\\ \mathrm{Subject}\;\mathrm{to}:\;\;F=KU\\ \;\;\;\;\;\;\;\;\;\;\;\;\;\;\;\;\;\;\;\;\;\;\;V\left(x\right)\leq V^{*}\\ \;\;\;\;\;\;\;\;\;\;\;\;\;\;\;\;\;\;\;\;\;\;\;0\leq x_{min}\leq x_{i}\leq 1\left(i=1,2,\cdots ,n\right) \end{array}\right.$$

where x is the relative density of the discretized elements; C(x), K, U, and F are the flexibility, overall stiffness matrix, displacement matrix, and external load matrix of the designable area, respectively; V(x) and $V^{*}$ are the actual volume of the designable area as a function of the variable x and the constrained volume required by the whole optimization problem, respectively; $x_{i}$ can vary continuously between $x_{min}$ and the maximum value of 1; and *i* is the number of elements.

### 2.3. BEGAN Theory

BEGAN is a robust and straightforward GAN architecture that achieves fast and stable convergence with standard training steps. It can generate high-quality images and control the balance between visual quality and image diversity. BEGAN is a milestone in the field of visual quality [34].

BEGAN uses the loss distribution derived from the Wasserstein distance to match the loss distribution of the self-encoder, intended to optimize a lower bound on the Wasserstein distance between the loss distributions of the autoencoder. To make the discriminator and generator balanced during the training process, a concept of equilibrium is proposed by introducing a hyperparameter γ∈[0,1]. γ is defined as:

(3) $$\gamma =\frac{E\left[L\left(G\left(z\right)\right)\right]}{E\left[L\left(x\right)\right]}$$

where E[L(G(z))] is the expectation of the loss of generated samples and E[L(x)] is the expectation of the loss of real samples. The discriminator has two goals, one is to self-encode real images and the other is to distinguish generated images from real images. γ can balance these two goals. Lower values of γ lead to lower image diversity because the discriminator is too concerned with self-encoding real images; hence, γ is referred to as the diversity ratio [35].

The objective function of BEGAN is:

(4) $$\left\{ \begin{array}{c} L_{D}=L(x)-k_{t}L(G((Z_{D}))\;\mathrm{for}\theta_{D}\\ L_{G}=L(G(Z_{G}))\;\mathrm{for}\theta_{G}\\ k_{t+1}=k_{t}+\lambda_{k}=(\gamma L(x)-L(G(Z_{G})))\mathrm{for}\mathrm{each}\mathrm{training}\mathrm{step}t \end{array}\right.$$

where the equilibrium E[L(G(z))]=γE[L(x)] is maintained using proportional control theory [36]. The variable $k_{t}\in \left[0,1\right]$ is used to control the degree of gradient decline emphasized during the use of $L\left(G\left(z_{D}\right)\right)$, initialized with $k_{0}=0$. $\lambda_{k}$ is the proportional gain of k, which is the learning rate of k. In essence, it can be considered as a closed-loop feedback control, where $k_{t}$ is adjusted at each step to maintain the equation $\gamma =\frac{E\left[L\left(G\left(z\right)\right)\right]}{E\left[L\left(x\right)\right]}$.

## 3. Intelligent Generation Method of Innovative Structures

The process of intelligently generating innovative structures includes building the dataset and generating structures by applying the BEGAN algorithm.

### 3.1. Building the Dataset

**Figure 2:**
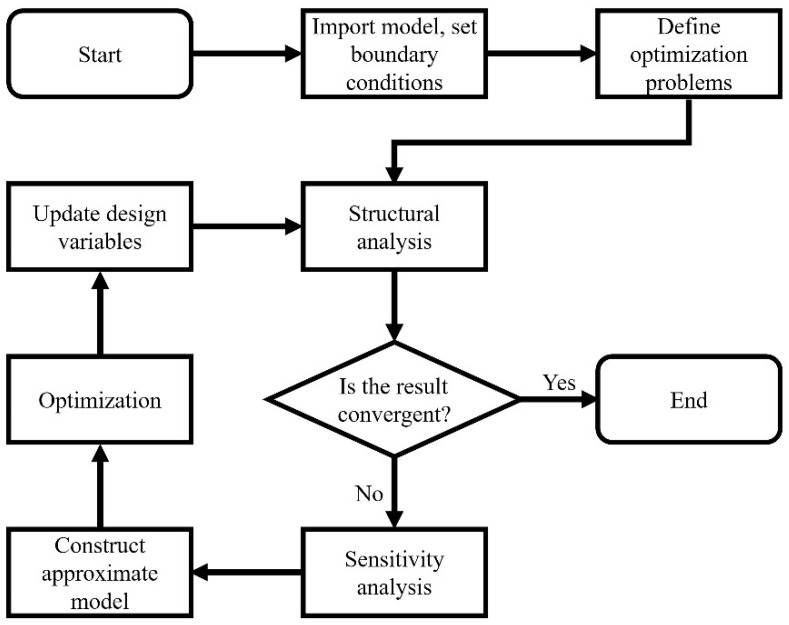
The process of optimization design.

To ensure the reliability of the dataset, the initial structural model was topologically optimized to obtain high-performance samples of deep learning. The initial model of the researched structure was first established, then it was imported into the optimization solver to retrieve topology optimization samples. The process of optimization design is shown in Figure 2.

Firstly, the finite element model and the boundary conditions were established. Then, the optimization objective and constraints were defined, and the design variables were initialized. The optimization solver performed a sensitivity analysis on partial derivatives of the design variables through design responses. The sensitivity information was then used to expand design responses for obtaining an explicit approximate model. The optimization solution is convergent until the difference between the objective values of two successive iterations is less than the given convergence tolerance.

By collecting images of topological results under different load cases, element density thresholds, manufacturing process constraints, and other optimization parameters, the dataset was built up. Then, the dataset was expanded by applying random rotation, vertical flip, horizontal flip, and other data enhancement methods, which not only inherit topology optimization features but also enhance the robustness of the deep learning model. After the dataset expansion process, the training set had a large number of images, which were divided into ten categories. The size of each image was 64 pixels × 64 pixels. Training set labels were defined as the corresponding types of load cases during topology optimization.

### 3.2. Generating Structures Applying the BEGAN Algorithm

The deep learning framework TensorFlow was used to build the BEGAN deep learning model. The BEGAN algorithm uses an adversarial training mechanism to learn topology optimization samples deeply and optimize the performance of the generated innovative structures.

The BEGAN algorithm was trained based on the machine learning platform TensorFlow 2.1. This platform is a flexible and comprehensive ecosystem of tools, libraries, and community resources that helps researchers to develop advanced machine learning techniques and facilitate developers to deploy applications that support machine learning. The highly encapsulated framework tf.keras was used to build the BEGAN deep learning model. The grayscale values of the input features were normalized to [0, 1] before training to make the networks converge faster [37,38].

The input of the BEGAN consisted of two parts. One was real images from the dataset, which was directly fed into the discriminator to obtain discriminative results. The other was the noise, which causes the generator to generate fake images. The noise of deep learning was sampled in a uniform distribution from −1 to 1, and its dimension was 100.

**Figure 3:**
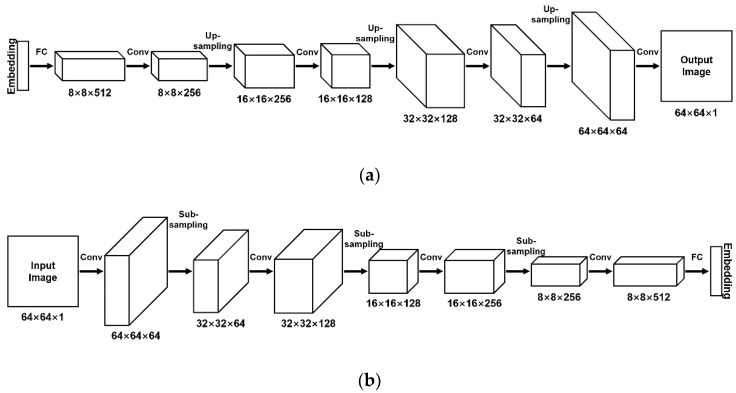
Network architecture for the generator and discriminator: (**a**) generator/decoder of the discriminator, (**b**) encoder of the discriminator.

The BEGAN algorithm uses an autoencoder as a discriminator and a decoder as a generator. Figure 3 shows network architecture for the generator and discriminator. The first layer of the decoder is a fully connected layer, while the rest of the structure consists of convolutional layers stacked with up-sampling layers. On the contrary, the front part of the encoder consists of convolutional layers stacked with sub-sampling layers, while the last layer is a fully connected layer. The input of the decoder is noise, while the output is a reconstructed image. The discriminator compresses the input image into a feature vector and then reconstructs the image from this vector [39].

In the generator, the last layer uses the tanh function as the activation function to expand the feature effect during the iteration when features differ significantly [40]. The remaining layers all use the exponential linear unit (ELU) activation function to add nonlinear factors to the deep learning model. The linear part on the right side enables the ELU to relieve the gradient disappearance, while the soft satiation on the left side allows the ELU to be more robust to input changes or noise. The output means of the ELU are close to zero; thus, the convergence speed is faster [41]. In addition to the last layer, the generator uses the batch normalization optimization algorithm to adjust the input features of each layer of the neural network to a standard normal distribution, which makes the data more concentrated without considering whether the data is too small or too large. Moreover, the batch normalization optimization algorithm can deal with the training problems caused by poor initialization, improve the stability of networks, and solve the problem of gradient disappearance in neural networks [42].

In the encoder of the discriminator, the ELU function and batch normalization are used for all layers except the last one. The adaptive moment estimation algorithm is used to optimize the loss functions of the generator and discriminator. The gradient’s first-order moment estimation and second-order moment estimation are combined to calculate the update step with the lowest memory requirements [43]. The hyperparameter learning rate of the Adam optimizer is 0.0002, which ensures the convergence speed of the network while avoiding the problems of model collapse caused by too large a learning rate, and slow convergence speed caused by too small a learning rate.

All images in the dataset were used as the training set to train the BEGAN deep learning model. The batch size was 64, which assured the diversity of training samples in each batch and prevented the gradient from falling into a local optimum solution due to the enormous batch size value. The diversity ratio γ was taken as 0.5 to guarantee the diversity and visual quality of the generated solution images, and to balance the optimization objectives of the generator and discriminator. The generated results were images of 30 design schemes arranged in 5 × 6. Loss function values and convergence measure values were extracted and visualized in each iteration.

## 4. Example 1: Baseplate of a Cast-Steel Support Joint

### 4.1. Engineering Background and Initial Model

**Figure 4:**
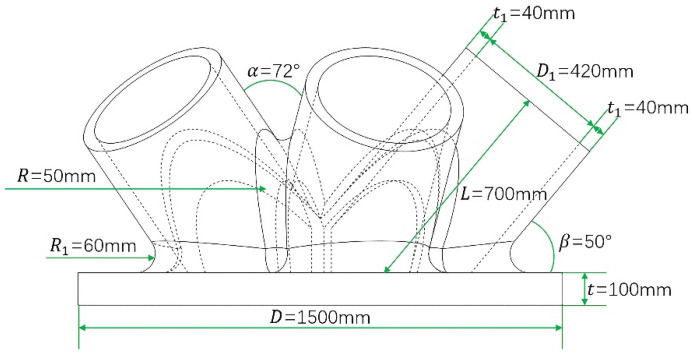
Geometric characteristics of the support joint.

**Figure 5:**
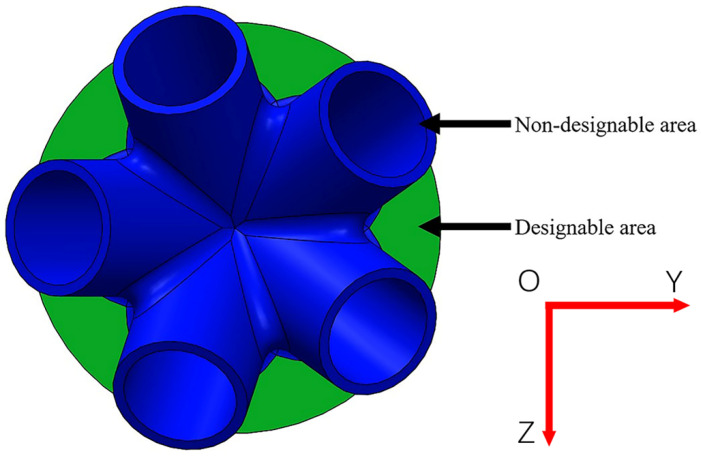
Initial support joint model.

Taking a cast-steel support joint in an actual project as the research object, the proposed method was applied for the intelligent generation of the baseplate. The cast-steel support joint’s initial model and geometric characteristics are shown in Figure 4 and Figure 5. Five branch pipes intersect at the baseplate of the support joint, through which the load is borne and transferred. Table 1 shows the geometric parameters of the support joint. The baseplate was a solid steel plate with a diameter of 1500 mm and thickness of 100 mm, its steel consumption was high, and there was great potential for optimization of the design. Hence, the method of this paper was used to intelligently generate innovative design schemes for the baseplate.

Vertical uniform loads were applied to each node on the top surface of the five branch pipes that were gridded. The total load of the five branch pipes is was 13,200 kN. Fixed end restraints were applied to all nodes on the bottom surface of the baseplate. Finally, the optimal design variables were defined as the element density in the baseplate area. The constraint was that the volume fraction of the baseplate area could not exceed 0.4, and the objective function was to maximize the stiffness.

### 4.2. Building the Dataset

**Figure 6:**
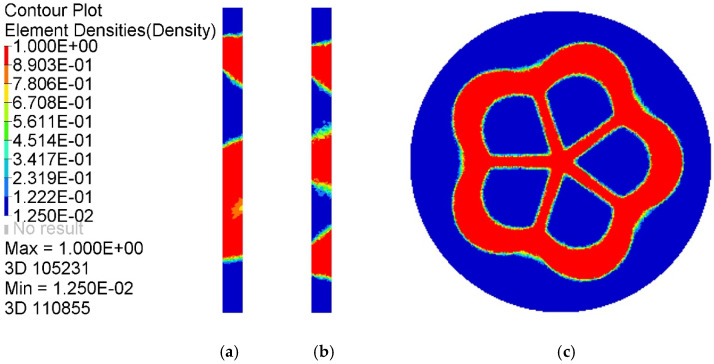
Static cloud maps of element density results: (**a**) X–Y midsection, (**b**) X–Z mid–section, (**c**) Y–Z midsection.

Topology optimization results of the baseplate were collected for building the dataset. Figure 6 shows static cloud maps of element density results in the optimized baseplate area. Under the given load case, the element density value near the connection between branch pipes and the baseplate tended to be 1, which is an essential part of the material distribution of the baseplate and must be retained during the optimization design process. The element density in the remaining areas was concentrated at 0.0125~0.1222, which can be selectively preserved according to the actual requirements during the optimization design process. In a comprehensive view, the baseplate area had a low material utilization rate and a significant potential for optimization.

**Figure 7:**
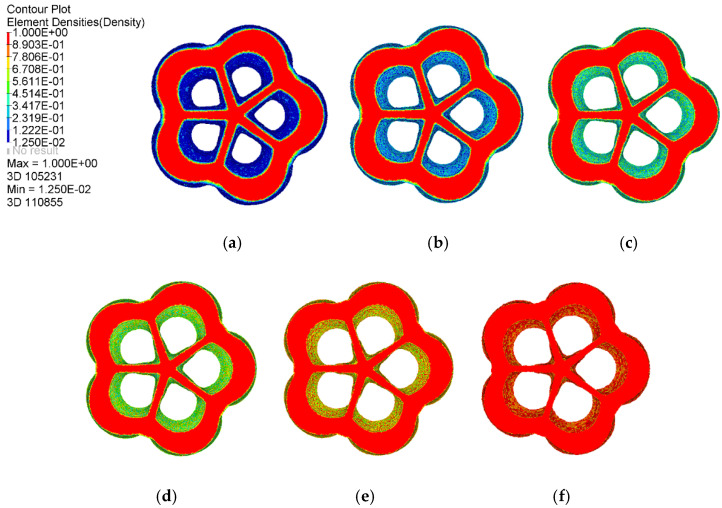
Iso–surface maps of element density results: (**a**) ρ=0.10, (**b**) ρ=0.25, (**c**) ρ=0.40, (**d**) ρ=0.55, (**e**) ρ=0.70, (**f**) ρ=0.85.

To find the optimal topological model of the baseplate and intuitively reflect the load transfer path in the baseplate area, the corresponding iso-surface maps of element density results when ρ=0.10~0.85 are shown in Figure 7. With an increase of the element density threshold, the topology optimization results showed an obvious material concentration tendency, a material accumulation phenomenon appeared near the intersection area between the branch pipes and the baseplate, the holes in the central area were further enlarged, and a large number of detailed connection materials were reduced or even disappeared. Geometric topology features were clear, and the retained material distribution reflected the force flow in the baseplate. When ρ=0.85, the red area of the baseplate was more uniformly distributed, redundant elements disappeared, and topological features were clear and reasonable; this can be regarded as an ideal baseplate optimization model.

**Figure 8:**
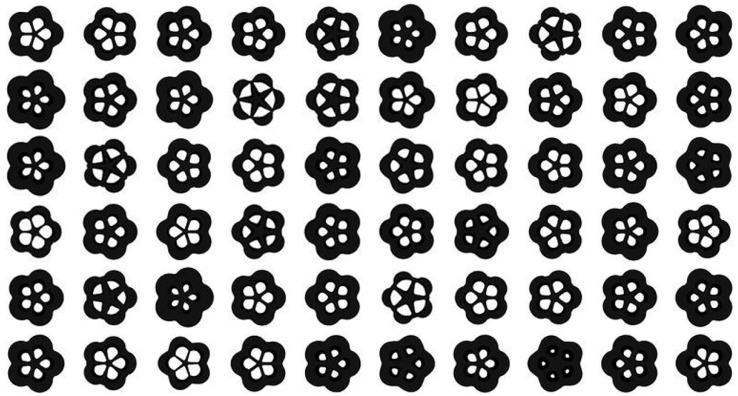
Some samples from the baseplate dataset.

After the analysis of the iso-surface maps of the element density results, the results of the baseplate under different parameters were selected to build the training dataset. There were 27,000 images in the training dataset that were divided into ten categories. Some samples are shown in Figure 8.

### 4.3. Generating Innovative Baseplates by BEGAN

**Figure 9:**
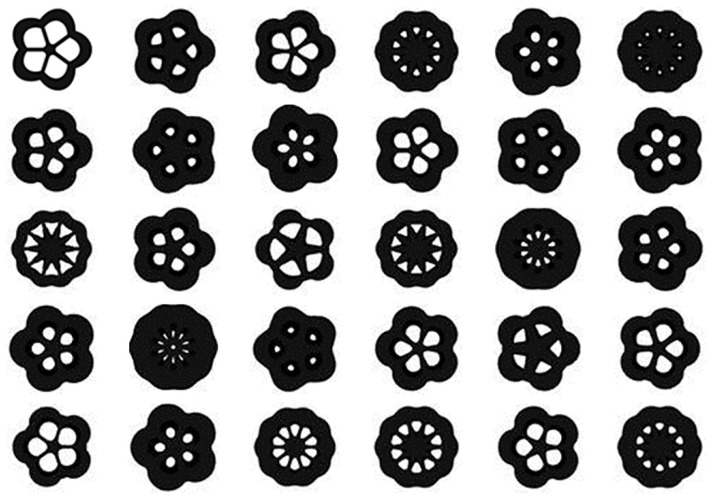
Thirty design schemes of the baseplate generated by BEGAN.

The dataset was fed into the BEGAN deep learning algorithm to generate the baseplate. Thirty design schemes of the baseplate generated by the BEGAN algorithm are shown in Figure 9.

The generated results in Figure 9 show that BEGAN could effectively extract samples’ features and generate a large number of innovative schemes for the baseplate. All the design schemes had good visual effects.

**Figure 10:**
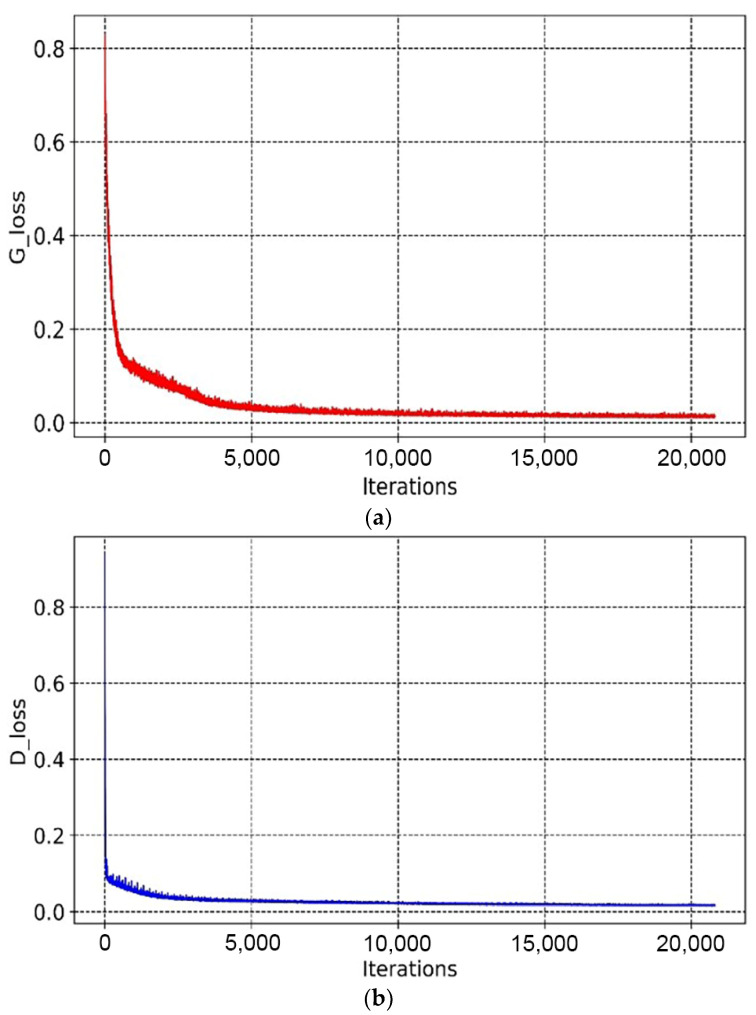
Variation curves of the loss function: (**a**) variation curve of the loss function of the generator, (**b**) variation curve of the loss function of the discriminator.

**Figure 11:**
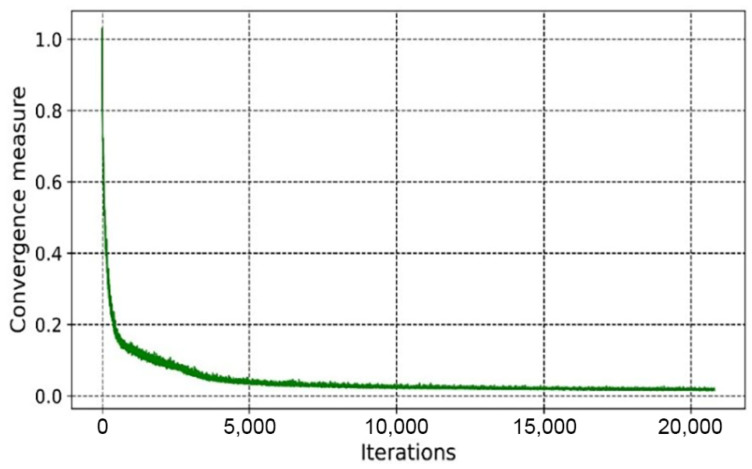
The global measure of convergence.

As shown in Figure 10 and Figure 11, the overall trends of the generator loss function, discriminator loss function, and global measure of convergence of BEGAN were decreasing, which indicates that the networks were converging with less oscillation during the training process. The fluctuation of the generator loss function is due to the random generation of images at the beginning, where the stochastic image reconstruction error is unexpected and fluctuates more. As the training proceeds, images generated by the generator converge to real images, and the reconstruction target is stable. Moreover, BEGAN can effectively avoid the problem of gradient vanishing during the training process and update the parameters of feature extraction networks in time to ensure the normal operation of the game confrontation process (i.e., it is of stable learning capability). Therefore, the BEGAN algorithm can generate innovative schemes that match the sample distribution of real baseplates. It has great potential in the field of intelligent generation for innovative structures.

In terms of time and memory consumption, although the intelligent generation method proposed in this paper uses topology optimization calculations to build the dataset, the topology optimization calculations of this model do not require the setting of detailed pre-processing parameters or the complex finite element iterative optimization process. Thus, the running speed can be significantly improved. The whole deep learning process only consumed 9.5 h to calculate 27,000 topological model features under hundreds of topology optimization parameters. This means that less than 1.3 s were consumed to compute a single model. In comparison, the duration for calculating the training data was five days. Hence, it is evident that the proposed method is computationally very efficient. Furthermore, the intelligent generation method can generate multiple baseplate models constantly, including various schemes with light mass and high stiffness, for evaluation.

### 4.4. Analysis and Evaluation of Schemes

An evaluation with four indexes, including innovation, aesthetics, machinability, and mechanical performance, was established to verify the feasibility and rationality of this generation method.

Given innovation, on the one hand, the generated baseplates were rich in style, the shapes of the holes were different, and the boundary transition of the holes was smooth. On the other hand, the material distribution of generated baseplates could inherit topology optimization features of the sample data. The material near the intersection between the baseplate and branch pipes was retained. The generated baseplates were innovative, and could provide designers with a variety of options.

From the perspective of aesthetics, the generated baseplates conformed to the aesthetics design concept of simplification and satisfied the principles of lightness, symmetry, and balance. Moreover, they had characteristics of smoothness and naturalness, which reflect harmony and beauty.

**Figure 12:**
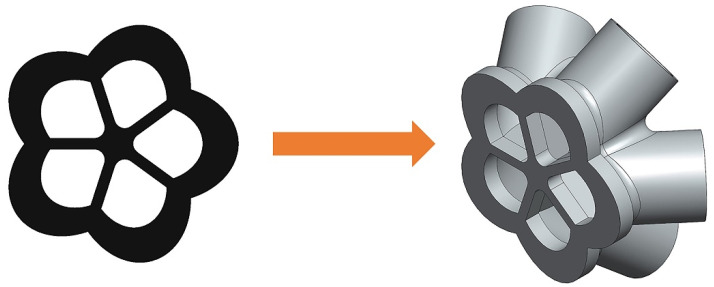
An example of a 3D support joint design using the selected 2D design.

Since material consumption is a significant economic indicator in engineering, this paper started by optimizing the material layout with the least material usage to meet the intended functional requirements. Thus, the lightest generated model was selected as the representative baseplate. Three-dimensional reconstruction technology was used to realize the 3D model reconstruction of the support joint by extracting image features of the generated baseplate and geometric characteristics of the initial 3D model [44], as shown in Figure 12.

**Figure 13:**
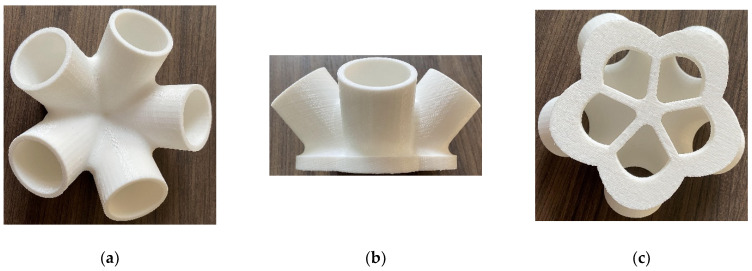
The joint model printed using FDM technology: (**a**) vertical view, (**b**) front view, (**c**) upward view.

To verify the manufacturing feasibility of the generated baseplates, a fused deposition modeling (FDM) 3D printer was used to fabricate the representative generated model. The material used was polylactic acid (PLA) plastic. First, the 3D model of the support joint was exported as an STL format file written in binary code through SolidWorks software. Then it was imported into Allcct slicing software to process the 3D model and translate the model file into a Gcode file that the 3D printer could read. Finally, the Gcode file was imported into the 3D printer to complete the filament feeding and equalization operations. When the equipment preheating is completed, the equipment begins to arrange consumables layer by layer to realize the solid transformation of the model. The joint model printed using FDM technology is shown in Figure 13.

From the printed solid model of the support joint, it can be seen that 3D printing technology can smoothly show the chamfered part of the joint and recover the complex detail with high smoothness and accuracy. Thus, the manufacture of the generated structures is feasible.

**Figure 14:**
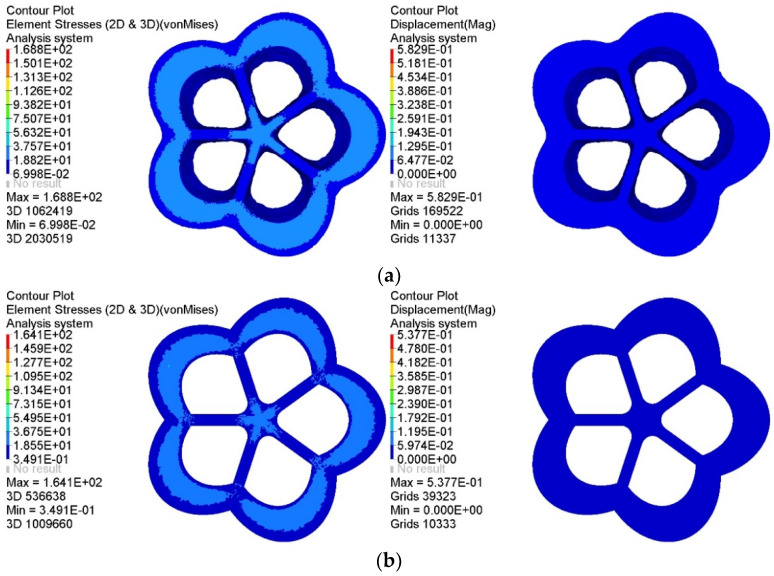
Comparison of static analysis results of two kinds of baseplates: (**a**) static analysis results of the topology optimization baseplate, (**b**) static analysis results of the intelligently generated lightest baseplate.

The representative baseplate was compared with the corresponding topology optimization baseplate to verify the mechanical performance of the generated baseplates. The static analysis results of the stress and displacement of each baseplate under the same load case are shown in Figure 14.

As shown in Figure 14, the mass of the topology optimization baseplate was 0.522 t, the maximum equivalent stress was 37.57 MPa, and the maximum displacement was 0.06477 mm, while the mass of the intelligently generated baseplate was 0.508 t, the maximum equivalent stress was 36.75 MPa, and the maximum displacement was 0.05974 mm. Compared with the topology optimization baseplate, the mechanical performance of the latter is further optimized through deep learning. Moreover, the stress concentration phenomenon was effectively reduced. The material distribution of the intelligently generated baseplate was biased towards the outer area of the intersection of branch pipes and the baseplate; thus, the distance between the outer material and the central material was larger, forming a more considerable stiffness to resist deformation and reduce displacement.

In a comprehensive view, the representative intelligently generated baseplate shows apparent advantages, with higher material utilization, uniform and reasonable displacement distribution, and excellent mechanical properties. It also had a reduced stress concentration existing at the intersection of the baseplate and branch pipes. The comparison results prove that the intelligent generation method can not only automatically generate innovative structures but also further optimize the material usage and mechanical performance of the structure.

## 5. Example 2: Cross Joint

### 5.1. Engineering Background and Initial Model

**Figure 15:**
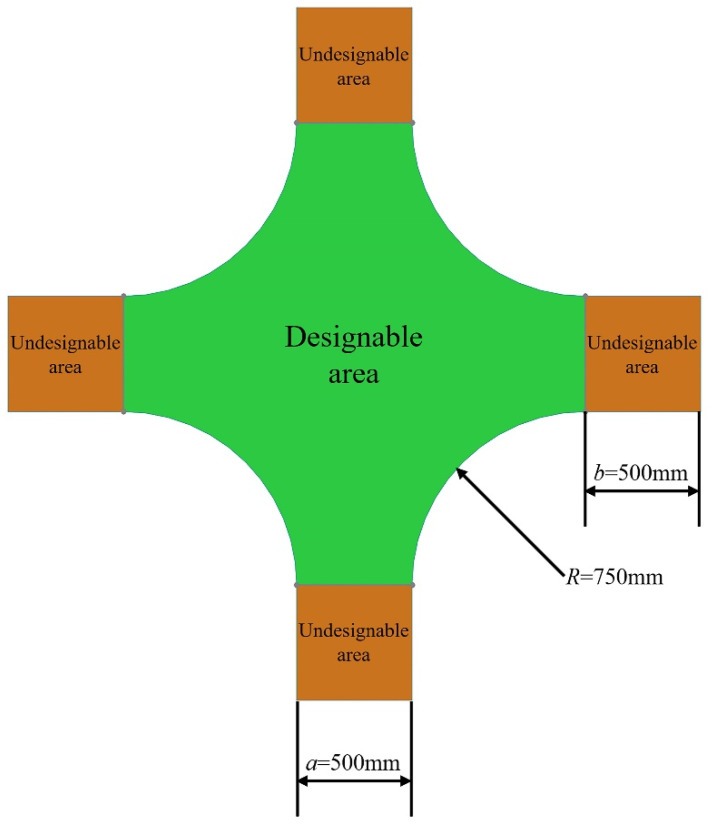
Geometric characteristics of the cross joint.

The initial model of a cross joint was created using SolidWorks software. Values of the joint geometric parameters are shown in Figure 15: the length of each branch was 500 mm, the width of each component was 500 mm, and the fillet radius between adjacent branch pipes was 750 mm. The whole model was divided into a designable area and four undesignable areas.

The bottom edge of the lower branch was subjected to a fixed end restraint. The ends of the remaining three branch pipes were each applied with a uniform linear load of 200 kN/m in the vertical direction. Design variables were defined as the element density of the designable area, and the constraint was that the volume fraction of the optimized region could not exceed 0.35.

### 5.2. Building the Dataset

**Figure 16:**
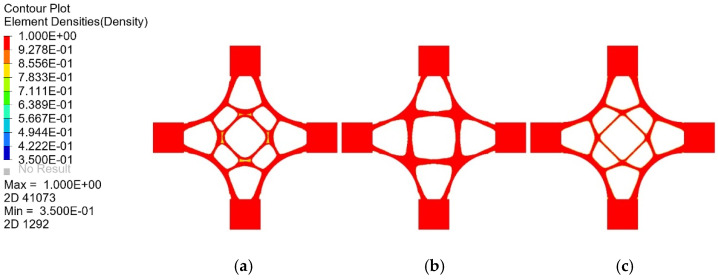
The influence of manufacturing process constraints on optimization results: (**a**) checkerboard control, (**b**) penalty factor, (**c**) symmetry constraint.

Applying various constraints on the actual manufacturing process, such as checkerboard control, penalty factor, and symmetry constraint eliminates the unnecessary load-transfer path in the optimization result. A more uniform material distribution is obtained, which facilitates the material flow in the casting process, provides sufficient stiffness for machining, and makes samples of the generated joints easy to manufacture. The influence of the manufacturing process constraints on the optimization results is shown in Figure 16.

**Figure 17:**
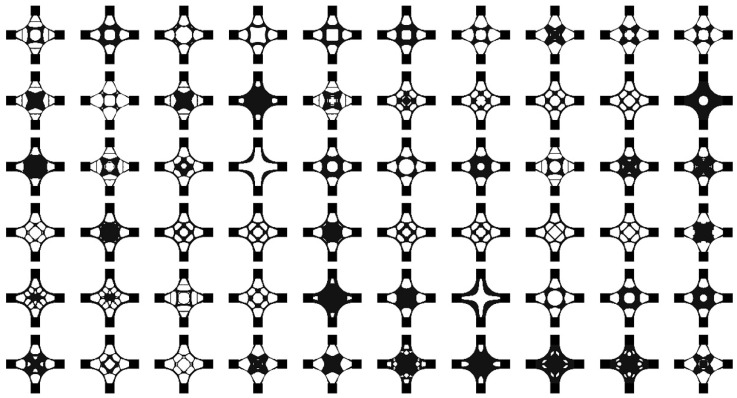
Some samples of the cross joint.

Samples generated by topology optimization were collected as images for the training set. Figure 17 shows some samples of the cross joint. Labels of the training set were defined to distinguish between different categories of load cases. This enabled the generation of joint images for specific load cases by specifying labels to realize the combination of deep learning and mechanics.

### 5.3. Generating Innovative Joints Using BEGAN

**Figure 18:**
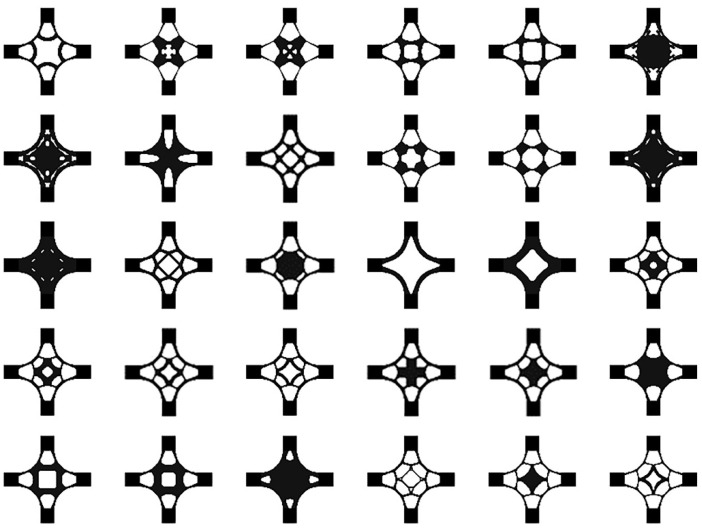
Thirty design schemes of the cross joint generated by BEGAN.

Using the balanced game of the generator and discriminator produces generated images that are increasingly closer to the input samples. Thirty design schemes of the cross joint generated by BEGAN are shown in Figure 18.

**Figure 19:**
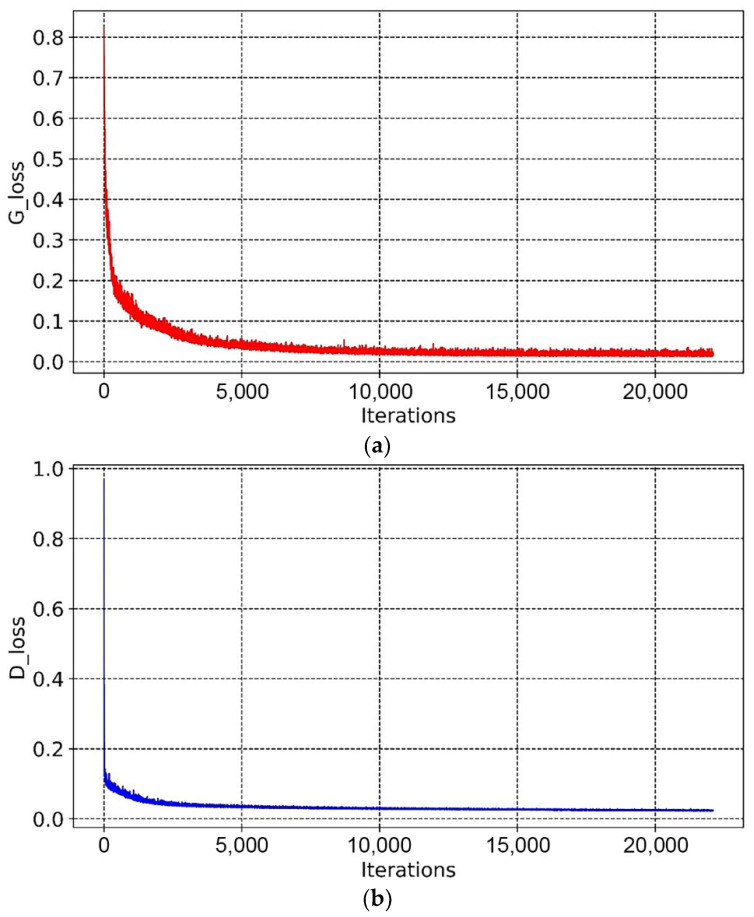
Variation curves of the loss functions: (**a**) variation curve of the loss function of the generator, (**b**) variation curve of the loss function of the discriminator.

**Figure 20:**
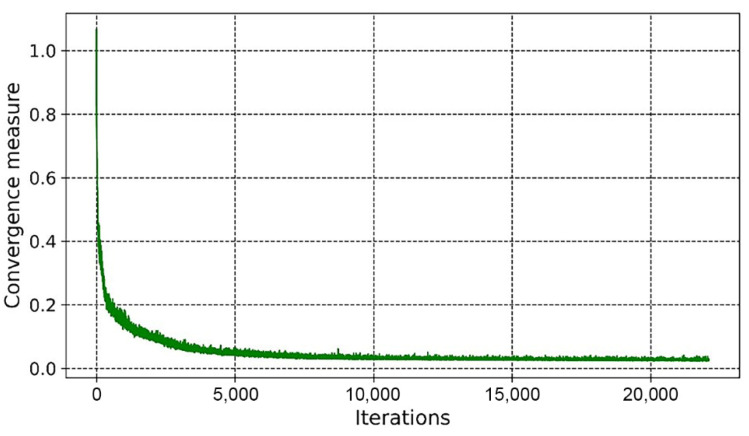
The global measure of convergence.

From Figure 19 and Figure 20, it can be seen that the overall trends of the generator’s loss function, discriminator’s loss function, and global measure of convergence of BEGAN converged steadily. The whole generation process consumed 6 h.

### 5.4. Analysis and Evaluation of Schemes

The generated design schemes were close to the samples in the dataset, but there were differences in the contour and material distribution characteristics, which are of innovation and good recognition. The overall style was soft and natural without distortion.

**Figure 21:**
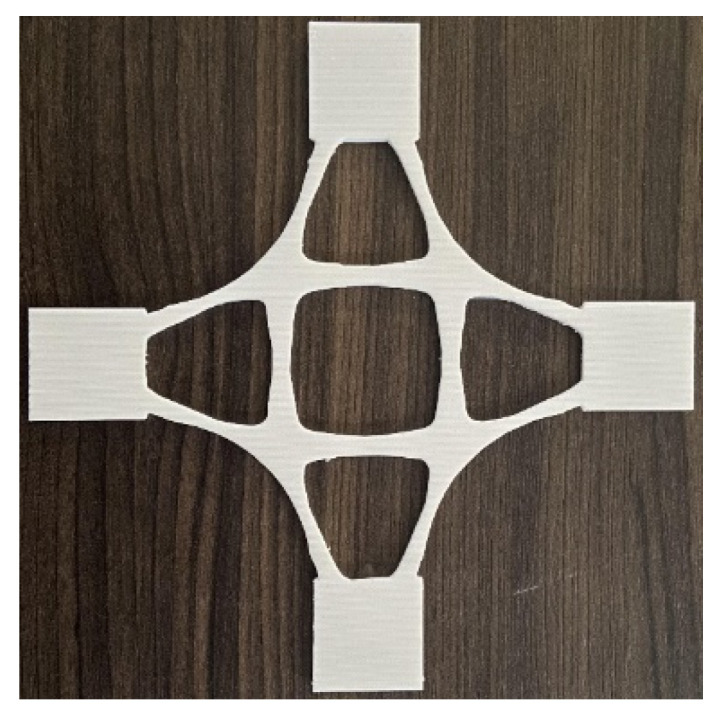
A representative generated model printed using FDM technology.

The material of the intelligently generated cross joints was coherent, and the main transmission path was clear and orderly. This prevents the formation of partial thin strips and ensures sufficient stiffness while facilitating the manufacture of joints. The boundary of the models was smoother, fuller, and symmetrical, and the material was evenly distributed, meeting the standards of aesthetics. Figure 21 shows a representative generated model printed using FDM technology. Additive manufacturing technology was valuable to produce the cross joint without any mold.

**Figure 22:**
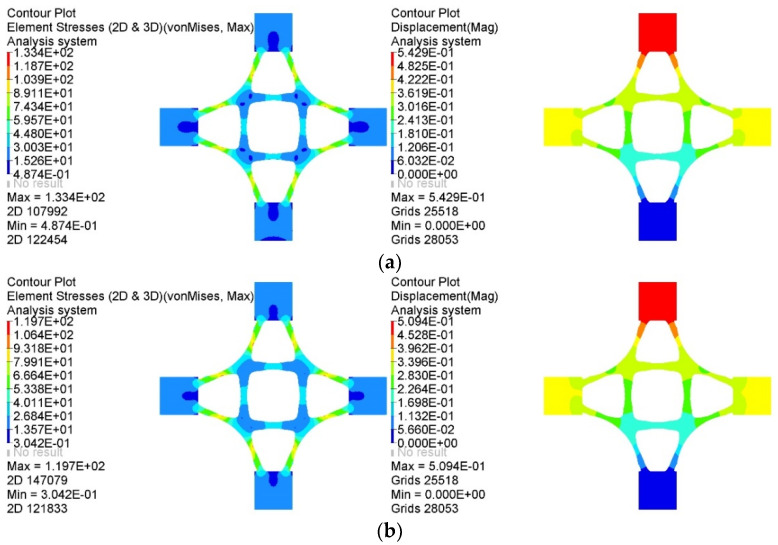
Static analysis results of different joints: (**a**) static analysis results of the topology optimization joint, (**b**) static analysis results of the representative intelligently generated lightest joint.

A comparison of the lightest representative intelligently generated joint with its corresponding topology optimization joint is shown in Figure 22. Not only can the intelligent generation method realize the goal of a lightweight joint, but the intelligently generated joint also has excellent mechanical performance.

## 6. Conclusions

In this paper, an intelligent generation method of innovative structures was proposed, and two generation cases systematically verified the feasibility of this method. The main conclusions are as follows:

(1) The traditional design method relies on design experience to build the initial model, and the computer only assists in computational analysis. In contrast, the intelligent generation method proposed in this paper can use the computer to automatically generate a large number of innovative structures with excellent mechanical performance. This method expands the scope of computer-aided design to intelligent structural generation, and designers will only need to select from several model schemes.
(2) It is feasible to obtain abundant topology optimization models under different parameters to provide excellent models for establishing deep learning datasets, which can not only solve the problem of building a model library with a large volume of data but also inherit the advantage of the high material utilization of topology optimization models.
(3) The BEGAN algorithm has a strong convergence and generation ability. It can solve the problems of gradient vanishing and poor quality of generated images encountered in traditional GAN algorithms and improve the robustness of the deep learning model.
(4) The intelligently generated structures are innovative and have good aesthetics, machinability, and mechanical performance that are reasonable and practical. With the development of cloud computing technology, the intelligent generation method can further improve the speed and quality of generated models.

## Figures and Tables

**Table 1:** Geometric parameters of the support joint.

Parameters	Values
Diameter of the baseplate/D(mm)	1500
Thickness of the baseplate/t(mm)	100
Length of branch pipes/L(mm)	700
Inside diameter of branch pipes/$D_{1}\left(\mathrm{mm}\right)$	420
Thickness of branch pipes/$t_{1}\left(\mathrm{mm}\right)$	40
Fillet radius between branch pipes/R(mm)	50
Angle between adjacent branch pipes/α(°)	72
Fillet radius between branch pipes and the baseplate/$R_{1}\left(\mathrm{mm}\right)$	60
Angle between branch pipes and the baseplate/β(°)	50

## Data Availability

Some or all of the data, models, and codes that support the findings of this study are available from the corresponding author upon reasonable request.
